# Resolution extension by image summing in serial femtosecond crystallography of two-dimensional membrane-protein crystals

**DOI:** 10.1107/S2052252517017043

**Published:** 2018-01-01

**Authors:** Cecilia M. Casadei, Ching-Ju Tsai, Anton Barty, Mark S. Hunter, Nadia A. Zatsepin, Celestino Padeste, Guido Capitani, W. Henry Benner, Sébastien Boutet, Stefan P. Hau-Riege, Christopher Kupitz, Marc Messerschmidt, John I. Ogren, Tom Pardini, Kenneth J. Rothschild, Leonardo Sala, Brent Segelke, Garth J. Williams, James E. Evans, Xiao-Dan Li, Matthew Coleman, Bill Pedrini, Matthias Frank

**Affiliations:** a Paul Scherrer Institute, 5232 Villigen PSI, Switzerland; b Lawrence Livermore National Laboratory, 7000 East Avenue, Livermore, CA 94550, USA; c Environmental Molecular Sciences Laboratory, Pacific Northwest National Laboratory, 3335 Innovation Boulevard, Richland, WA 99354, USA; d Arizona State University, 300 East University Drive, Tempe, AZ 85287, USA; e Center for Free-Electron Laser Science, DESY, Notkestrasse 85, 22607 Hamburg, Germany; f Linac Coherent Light Source, 2575 Sand Hill Road, Menlo Park, CA 94025, USA; g Physics Departement, Boston University, 590 Commonwealth Avenue, Boston, MA 02215, USA; h National Science Foundation BioXFEL Science and Technology Center, 700 Ellicott Street, Buffalo, NY 14203, USA

**Keywords:** serial crystallography, free-electron lasers, membrane proteins, two-dimensional crystals

## Abstract

Previous proof-of-concept measurements on single-layer two-dimensional membrane-protein crystals performed at X-ray free-electron lasers (FELs) have demonstrated that the collection of meaningful diffraction patterns, which is not possible at synchrotrons because of radiation-damage issues, is feasible. Here, the results obtained from the analysis of a thousand single-shot, room-temperature X-ray FEL diffraction images from two-dimensional crystals of a bacteriorhodopsin mutant are reported in detail. The high redundancy in the measurements boosts the intensity signal-to-noise ratio, so that the values of the diffracted intensities can be reliably determined down to the detector-edge resolution of 4 Å. The results show that two-dimensional serial crystallography at X-ray FELs is a suitable method to study membrane proteins to near-atomic length scales at ambient temperature. The method presented here can be extended to pump–probe studies of optically triggered structural changes on submillisecond timescales in two-dimensional crystals, which allow functionally relevant large-scale motions that may be quenched in three-dimensional crystals.

## Abstract 2

The resolution limit of serial diffraction from two-dimensional crystals at a free-electron laser was extended to the detector edge (4 Å) by exploiting the large redundancy of the data set.

## 1. Introduction   

X-ray diffraction is one of the most prominent methods used to investigate the structure of biological molecules, as witnessed by the huge number of structures deposited in open-access databases in recent decades. Many of the challenges have been related to the fight against radiation damage, which limits the minimal size of the three-dimensional crystals that can be measured. Continuous progress has now made it possible to collect data from crystals as small as few micrometres at synchrotron sources. Recently, data collection without cryogenic protection of the samples has been demonstrated at synchrotrons (Botha *et al.*, 2015 ▸; Nogly *et al.*, 2015 ▸; Martin-Garcia *et al.*, 2017 ▸), which opens the way to time-resolved studies on millisecond timescales that require physiological temperature conditions.

X-ray free-electron lasers (X-ray FELs; Emma *et al.*, 2010 ▸; Pile, 2011 ▸) allow the extension of X-ray crystallography towards even smaller crystals, down to the submicrometre range. Radiation damage is overcome *via* ultra-intense and ultrashort X-ray pulses, with the data being collected in the diffraction-before-destruction mode (Boutet *et al.*, 2012 ▸). This has made it possible to address very delicate, challenging protein targets (Liu *et al.*, 2013 ▸; Zhang *et al.*, 2015 ▸, 2017 ▸) with X-ray FELs. Room-temperature measurements are permitted, and accessing the femtosecond time scale is possible *via* pump–probe experiments, which are typically triggered by an external laser source (Kern *et al.*, 2013 ▸; Tenboer *et al.*, 2014 ▸; Pande *et al.*, 2016 ▸; Young *et al.*, 2016 ▸; Suga *et al.*, 2017 ▸; Aquila *et al.*, 2012 ▸).

Even more challenging than three-dimensional nanocrystals are two-dimensional crystals, which consist of a periodic arrangement of molecules in a two-dimensional layer. This state of aggregation is of interest, especially in the case of membrane proteins, because it may better reproduce the conditions that occur within the cell membrane (Fujiyoshi, 2011 ▸). Furthermore, the all-important structural changes induced by external stimuli (Rosenbaum *et al.*, 2009 ▸; Deupi *et al.*, 2012 ▸) are expected to follow the natural dynamics, being less hindered by the steric contacts from molecules in the neighbouring layers than in a three-dimensional crystal. The X-ray diffraction power of two-dimensional protein crystals is orders of magnitude smaller than that of their three-dimensional counterparts because they consist of one (or a few) molecule layers and because the signal is spread over one-dimensional Bragg rods instead of being concentrated in Bragg spots. Therefore, before the era of X-ray FELs, two-dimensional membrane-protein crystals could only be studied successfully at high resolution by electron microscopy or diffraction (Unwin & Henderson, 1975*a* ▸,*b* ▸; Ceska & Henderson, 1990 ▸; Kunji *et al.*, 2000 ▸; Kühlbrandt *et al.*, 1994 ▸; Schertler *et al.*, 1993 ▸; Henderson *et al.*, 1990 ▸; Gonen *et al.*, 2005 ▸). The benefit of using electrons resides in the observation that the ratio between elastic scattering and damaging absorption events is substantially more favourable compared with X-rays (Henderson, 1995 ▸).

With the advent of X-ray FELs, dynamical studies with unprecedented time resolution became possible using three-dimensional protein crystals. From this perspective, data collection from two-dimensional crystals at X-ray FELs in a serial femtosecond crystallo­graphy (SFX) mode was explored. During initial beamtime at the Coherent Diffraction Imaging (CXI) endstation of the Linac Coherent Light Source (LCLS) in May 2012 we collected, to our knowledge, the first ever two-dimensional crystal X-ray diffraction patterns in transmission. The crucial improvement with respect to previous unsuccessful attempts was to focus the beam down to several hundred nanometres, tailored to the typical size of a two-dimensional crystal. Thus, it was proven that the available X-ray flux at CXI is sufficient to counteract the extremely weak diffraction power (Frank *et al.*, 2014 ▸). Later, in May 2013, better sample-preparation and delivery methods allowed a dozen indexable diffraction images to be recorded from single two-dimensional crystals of bacterio­rhodpsin, exhibiting clear signals up to 7 Å resolution (Pedrini *et al.*, 2014 ▸).

In November 2013 a third CXI beamtime took place, devoted to exhaustive investigations of two-dimensional crystals of different proteins prepared on various supports and following different protocols. The remarkable improvements in the data-collection automation at CXI allowed larger data sets to be recorded than previously possible in a few shifts of beamtime, which was the key to boosting the quality of the data-analysis outcome. We report here on the results obtained from a data set of about 1000 images recorded from two-dimensional crystals of a bacteriorhodopsin mutant (bR-D96N). The data set was collected in the ‘untilted’ configuration, meaning that the X-ray beam perpendicularly hit the membrane on which the two-dimensional crystals were deposited. We explain in detail the protocol used to identify and index the diffraction patterns, as used in part in a previous publication (Pedrini *et al.*, 2014 ▸) but since upgraded to handle patterns containing multiple lattices. The huge redundancy in the observation of each reflection was exploited by suitably adding up images to enhance the Bragg peak signals and in parallel obtain a much more homogeneous background. Thus, peaks to a resolution of at least 4 Å (corresponding to the detector edge) are clearly visualized, and their intensity is determined in a reliable manner with a signal-to-noise ratio of above 7 in the highest resolution bin.

This represents a proof-of-principle study, in which we show that the intrinsic limitation in the signal-to-noise ratio of reflections from monolayers can be efficiently dealt with thanks to the high redundancy of the data, and that this allows the resolution limit of the experiment to be extended. In §[Sec sec4]4, we briefly address the other key aspect of reconstructing the intensity in three-dimensional reciprocal space and demonstrating that it encodes useful structural information, which will be the subject of a future article.

## 2. Methods   

### 2.1. Sample preparation   

Purple membrane was isolated from *Halobacterium salinarum* expressing the mutant gene for bR-D96N, and detergent-stabilized two-dimensional crystal solutions were prepared using previously described procedures (Frank *et al.*, 2014 ▸; Pedrini *et al.*, 2014 ▸). The two-dimensional crystals were washed with water and suspended in 0.5%(*w*/*v*) glucose to a final protein concentration of 0.5 mg ml^−1^ just before application onto the sample carrier for X-ray diffraction data collection.

**Figure 1:**
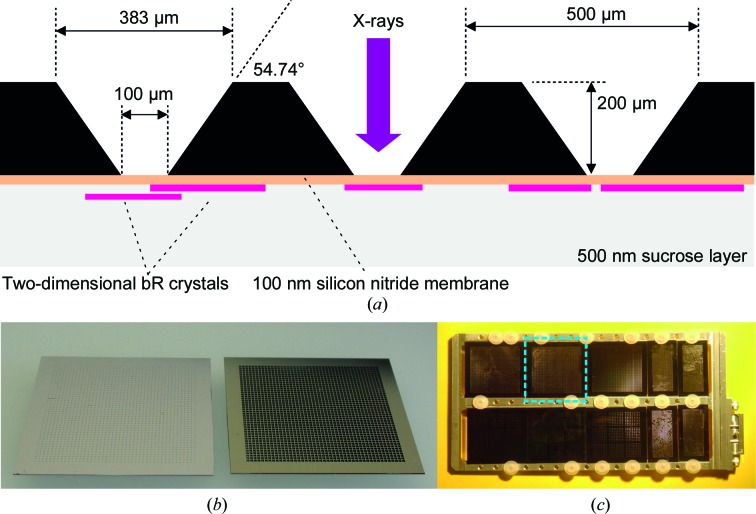
Sample support. (*a*) Sketch of the chip carrying the two-dimensional bR-D96N crystals (the membrane thickness is not to scale). (*b*) Picture showing the two faces of the chip, with the face encountered by the incoming X-ray beam on the right. (*c*) Picture of various chips fixed on the metallic frame that was mounted on the translation stages inside the CXI vacuum chamber for X-­ray diffraction data collection. The chip carrying the two-dimensional bR-D96N crystals is delimited by the dashed blue line.

A silicon chip with an area of 25 × 25 mm and 200 µm thickness, produced by Silson Inc., was used as a carrier. The chip had a 44 × 44 array of 100 × 100 µm windows made of a 20 nm thick Si_3_N_4_ membrane (Figs. 1 ▸ *a* and 1 ▸ *b*). A total of about 30 µl of two-dimensional bR-D96N crystal suspension was deposited onto the silicon chip and allowed to dry in air. The resulting glucose layer served to protect the protein sample from dehydration in the experimental chamber vacuum.

### 2.2. Experimental setup and data collection   

X-ray diffraction measurements were carried out at the CXI experimental station (Liang *et al.*, 2015 ▸) of the LCLS using the 0.1 µm focus setup. The photon energy was set to 8.5 keV (1.5 Å). The beam size was estimated to be below 200 nm FWHM. The pulse energy was ∼2 mJ and the pulse length was ∼35 fs.

The chip covered with two-dimensional bR-D96N crystals was mounted, together with other sample supports, on a metallic frame that was fixed to the sample stages inside the experimental chamber (Fig. 1 ▸ *c*). Data collection was performed in a vacuum environment, whereby all of the chips on the frame were measured within about 6 h. The sample stages were scanned in steps, membrane by membrane and row by row, with X-ray pulses initiated on-demand (LCLS burst mode) at a rate of about 1.5 per second in order to hit the Si_3_N_4_ windows. The silicon frame was kept perpendicular to the X-ray beam, which we call the untilted data-collection configuration. Diffraction patterns were recorded using the Cornell–SLAC pixel-array detector (CSPAD) with 2.3 megapixels of 110 µm in size, which was placed 235 mm downstream of the sample in the same vacuum chamber (Blaj *et al.*, 2015 ▸). The final data set discussed here consisted of 968 images. These were summed, and an initial detector geometry and an overall approximate direct-beam position were determined from the powder rings obtained.

### 2.3. Data analysis   

We denote the reciprocal-lattice basis vectors of the two-dimensional crystal as a* and b*, and the unit vector perpendicular to the plane spanned by a* and b* as 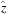
*. The two-dimensional periodic arrangement in real space results in reciprocal-space structure factors that are nonvanishing only along the Bragg rods. These are lines labelled by two integer indices (*h*, *k*) parametrized as 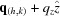
*; the continuous parameter *q_z_* is the out-of-plane momentum transfer. The in-plane momentum transfer associated with the rod is **q**_(*h*,*k*)_ = *h* a* + *k* b*, and we denote *q* = |**q**|. In a diffraction image, the high-intensity spots, which we call Bragg peaks in analogy to the nomenclature used in three-dimensional crystallography, are observed in directions corresponding to the intersection of the Ewald sphere, fixed by the direction and photon energy of the incoming X-rays, with the Bragg rods (Supplementary Fig. S1). A Bragg spot is labelled by [*h*, *k*, *q_z_*(*h*, *k*, φ, η)], where *q_z_* depends on the tilt angle η and the orientation angle φ of the crystal on the sample-support plane. In the untilted data-collection configuration, however, *q_z_* depends only on the two rod indices (*h*, *k*), so that for simplicity we use these two indices to label a Bragg peak.

**Figure 2:**
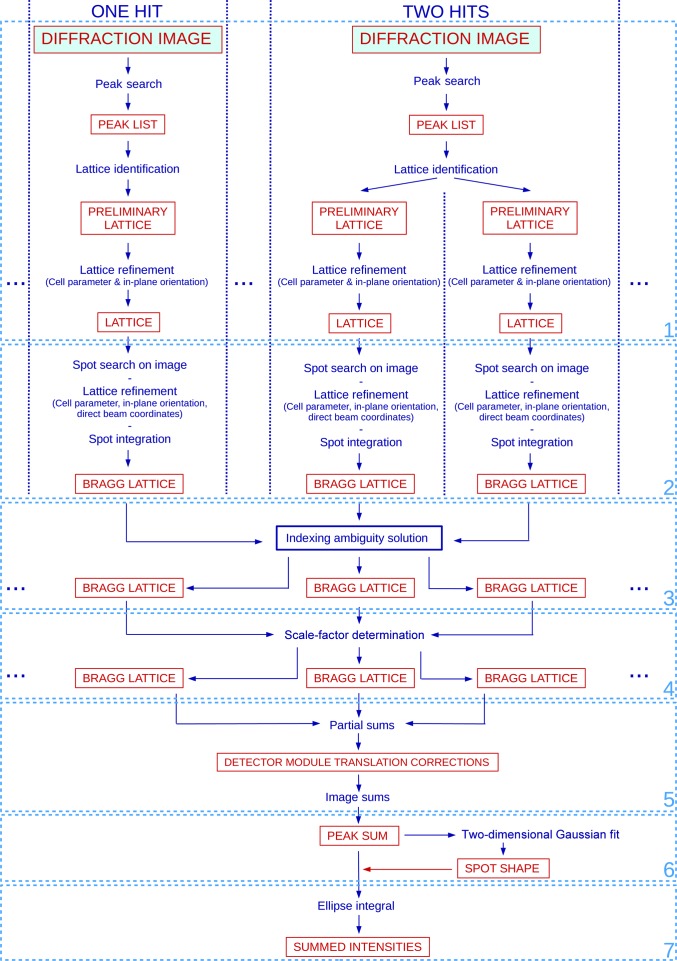
Data-analysis protocol. The flowchart shows an overview of the applied data-analysis protocol. The various steps are described in §[Sec sec2.3]2.3. The columns with dotted-line borders correspond to one diffraction image, from which one or more lattices are identified.

The entire analysis of the collected diffraction images was carried out under the assumption of *p*_3_ symmetry of the two-dimensional real-space lattice (plane group 13; Henderson *et al.*, 1990 ▸). Therefore, we have *a* = *b*, and the angle between the two unit-cell vectors is 2π/3. The data-analysis pipeline consists of seven subsequent steps, schematized in Fig. 2 ▸ and explained in more detail below. Unless specified differently, the processing was performed using scripts written in the Python 2.7 language. The procedure delivers a list of Bragg reflection intensities up to the highest possible resolution, as well as quality indicators for the intensities.

#### 2.3.1. Step 1: lattice identification   

The diffraction images were processed with *Cheetah* (Barty *et al.*, 2014 ▸) to convert the data format from XTC to HDF5, apply dark-current and gain-calibration corrections, and produce a list of high-intensity spot coordinates. These peaks were then arranged, if meaningful, into groups compatible with diffraction patterns from single two-dimensional crystals with lattice parameter *a* = 62.45 Å, as known from previous studies (Henderson *et al.*, 1990 ▸), with the X-ray photon energy associated with that measurement. We associated a preliminary lattice with each group with at least 20 peaks, with the orientation in the sample plane parametrized by the angle φ. This method allowed the identification of up to five independent lattices per image.

#### 2.3.2. Step 2: lattice refinement   

The parametrization of each lattice was then refined further, relying on the experimental intensities in the corresponding CSPAD diffraction pattern. For all of the peaks (*h*, *k*) up to 4 Å in-plane resolution, which corresponds to the detector edge, an image sector consisting of 96 × 96 detector pixels centred at the position predicted from the previously determined lattice orientation was extracted. In each sector, the background area was defined as the union of regions characterized by low fluctuations in the intensity within the detector module to which the predicted peak position belongs. The intensity in the background area was fitted with a tilted plane, which was then subtracted from the experimental intensities in the sector. Afterwards, a connected region of high-intensity pixels was searched, and if identified its centre-of-mass position was defined as the experimental position of the peak. Lattices with less than 28% of the peaks identified were discarded. The experimental positions of the peaks served as the input for the refinement routine. In addition to the mentioned parameters *a* and φ, it turned out to be convenient to also refine the co­ordinates of the direct-beam position on the detector. The routine consisted of an iterative application of either systematic grid searches or the Powell algorithm (Powell, 1964 ▸).

Once the four parameters of a lattice had been determined, the position of each peak in its corresponding sector was calculated and the peak area was defined as a circle of radius five pixels centred at the peak position. If the area belonged entirely to one detector module, then the intensity *I*(*h*, *k*) of the peak was calculated by integration of the intensities over the pixels in the circle.

#### 2.3.3. Step 3: indexing-ambiguity solution   

Lattice indexing in space group *p*_3_ is affected by ambiguity in the assignment of indices. Indeed, the physical operations of reversing the face of the crystal or rotating the crystal in plane by an angle π around the axis perpendicular to the crystal plane do not modify the peak positions, but modify the indices assigned to each peak. In the untilted experimental configuration, the following operations have to be considered.

(i) *T*_0_: (*h*, *k*) → (*h*, *k*) (identity).
(ii) *T*_1_: (*h*, *k*) → (−*h*, −*k*) (in-plane π rotation).
(iii) *T*_2_: (*h*, *k*) → (−*k*, −*h*) (face reversal).
(iv) *T*_3_: (*h*, *k*) → (*k*, *h*) (in-plane π rotation followed by face reversal).

To resolve the indexing ambiguity, the problem is to associate with each lattice *L* one of the four transformations *T_L_* that acts on the measured reflection intensities as 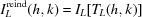
in such a way that the correlation of the transformed intensities of equivalent peaks in different lattices is maximal. Only peaks up to 7 Å in-plane resolution were considered. Two alternative methods were used. The first, based on the evaluation of intensity correlations from pairs of lattices, is described in Appendix *A* [App appa]. The second method uses the expansion–maximization–compression (EMC) algorithm, which was first proposed to orient weak diffraction patterns from single molecules (Loh & Elser, 2009 ▸) and was later applied to three-dimensional crystallography. Our implementation to two-dimensional crystallographic data is described in Appendix *B* [App appb]. The per-lattice transformations *T_L_* obtained from the two methods were compared to check for self-consistency.

#### 2.3.4. Step 4: lattice scaling   

To compensate for variations in the crystal area exposed to the X-rays and for fluctuations in the X-ray pulse energy, a lattice-dependent scaling of the intensities, 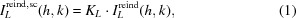
was determined for best matching of the equivalent reflection intensities up to 7 Å in-plane resolution in the data set, and such that the average of all scaling factors *K_L_* is unity. The procedure is explained in detail in Appendix *C* [App appc]. Lattices that could not be scaled were discarded.

#### 2.3.5. Step 5: signal-to-noise enhancement by image sums   

Many of the diffraction peaks at higher than 7 Å in-plane resolution turned out to be very weak and almost hidden in the noise. The signal-to-noise ratio of the reflection intensity is enhanced by measuring the same peak many times. Two conceptually equivalent methods are possible. In the first, which is the standard in protein three-dimensional crystallo­graphy both at synchrotrons and X-ray FELs, the intensity is measured in each image separately and the final intensity of a reflection is obtained as the average of the intensities of equivalent peaks. In the second, the pixel intensities of equivalent sectors of each image are summed, and the final intensity of the reflection is extracted from the obtained image sum. We followed the second path because of some key advantages: the background subtraction turned out to be remarkably more reliable, the integration area could be defined consistently, and in parallel small errors in the geometry of the outer detector modules could be identified and corrected.

The intensity in an image region around a predicted position of a peak in the *p*_3_ reflection class {(*h*, *k*)} was first rescaled with the lattice-specific scale factor of (1), and then interpolated linearly on a 50 × 50 point grid centred at the predicted position, with the *x* and *y* axes in radial and azimuthal directions with respect to the direct-beam position, respectively, and with a pitch corresponding to one detector pixel. The intensity pattern 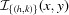 associated with the reflection {(*h*, *k*)} was then obtained as the per-pixel average over the *N*_{(*h*,*k*)}_ equivalent observations, followed by a background-subtraction procedure analogous to that described previously in §[Sec sec2.3.2]2.3.2. Because of the assumed *p*_3_ symmetry, each lattice can provide up to three equivalent observations of a reflection.

#### 2.3.6. Step 6: reflection-area determination   

The intensity array 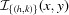 in each reflection-image sum was fitted with a two-dimensional Gaussian function 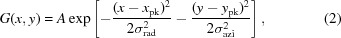
with adjustable parameters *A*, *x*_pk_, *y*_pk_, σ_rad_ and σ_azi_.

In a first attempt, the behaviour of the radial and azimuthal widths σ_rad_ and σ_azi_ as a function of the in-plane momentum transfer *q* exhibited a step-like feature at 7–6 Å resolution (Supplementary Fig. S2). Since the detector geometry was first set based on the powder rings, the intensity of which drops markedly at this resolution, we suspected that the origin of the hump relied on small errors in the geometry of the outer modules. We therefore optimized the detector geometry using the procedure described in Appendix *D* [App appd].

After determination of the outer module geometry corrections, the image-summing procedure and the subsequent peak-shape fitting were repeated. The step-like feature in the widths σ_rad_ and σ_azi_ disappeared, and their behaviour could be modelled with the polynomial function 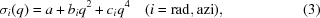
which describes the spot shape as a function of the in-plane momentum transfer. The reflection area was then defined as an elliptical region 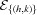 centred at the predicted peak position with semi-axes 2.5σ_rad_(*q*_*h*,*k*_) and 2.5σ_azi_(*q*_*h*,*k*_).

#### 2.3.7. Step 7: reflection-intensity determination   

The final reflection intensities were obtained by integration over the ellipse area, 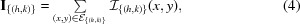
and correspond to the number of photons scattered on average by a two-dimensional crystal into the Bragg peak {(*h*, *k*)}.

#### 2.3.8. Data-quality evaluation   

As an initial data-quality indicator, we considered the signal-to-noise ratio S/N, which for each reflection is given by 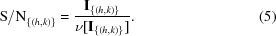
The noise was calculated according to the formula 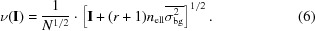
The first term in the square root accounts for the intrinsic Poisson noise, while the second accounts for noise effects in the integration and in the determination of the background level. The parameter *r* is the ratio between the elliptical integration area and the area used to establish the background, *n*_ell_ is the number of detector pixels in the elliptical integration area and 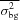 is the variance of the intensity in the background areas of single image sectors, averaged over all sectors used to build the image sum 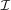. (6) was inverted to estimate the number of observations required with the same setup to achieve an S/N of unity, 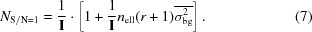
As a second indicator, we used the split correlation coefficient CC_1/2_. For each reflection, the peak observations were split into two sets, within which the image sums and the reflection intensities were calculated in the same manner as for the non-split data. The linear correlation between reflection intensities in resolution bins of 22 reflections were calculated. The final per-bin CC_1/2_ values were then computed as the average of the correlations obtained from ten different random splittings of the peak observations.

## 3. Results   

**Figure 3:**
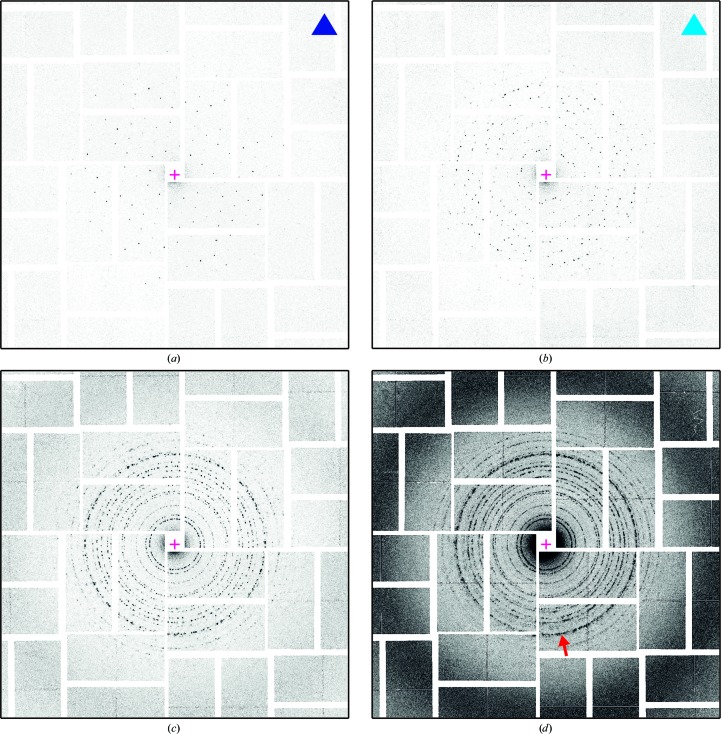
Diffraction images. Examples of the different types of collected diffraction images. (*a*) Single lattice. (*b*) Few lattices. (*c*) Multiple lattices. (*d*) Powder-like. The intensity scale is the same in all panels. Images of types (*a*) and (*b*) are indexable. In (*d*) the high-intensity ring labelled by the red arrow corresponds to the (3, 4) reflection and is at 8.9 Å in-plane resolution. The magenta cross represents the direct-beam position.

Of the 968 collected images, 410 contained indexable diffraction patterns, either from a single lattice (Fig. 3 ▸ *a*) or from a few lattices (Fig. 3 ▸ *b*). The other images contained many patterns, were powder-like (Figs. 3 ▸ *c* and 3 ▸ *d*), contained no signal arising from two-dimensional crystals or were blanks (no X-ray pulse hit the sample).

**Figure 4:**
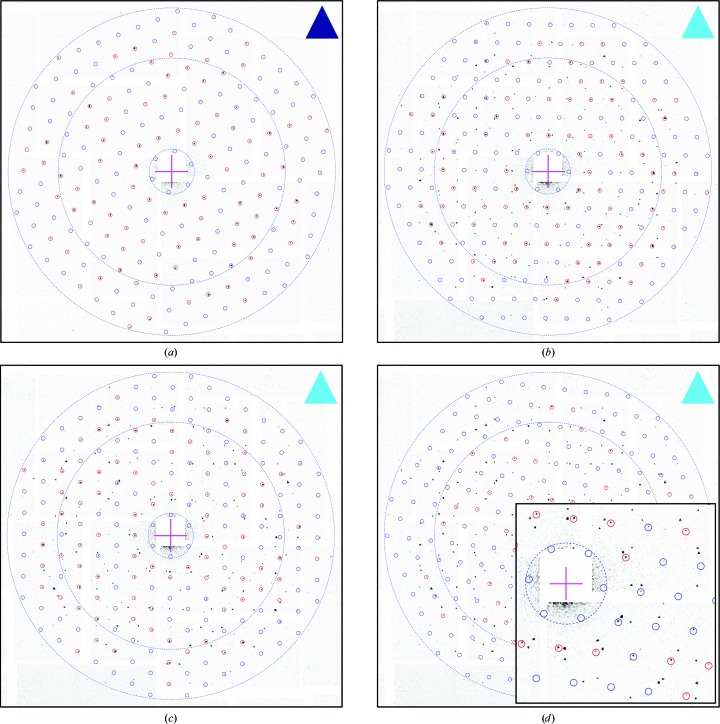
Lattice patterns. The four plots show examples of lattices obtained after step 2 of the data-analysis procedure, and are represented as circles that mark the expected peak positions down to an in-plane resolution of 7 Å, superimposed on the corresponding diffraction image. Red circles indicate the more prominent peaks that were identified and used to establish the precise lattice orientation and its unit-cell size. (*a*) is the single lattice image of Fig. 3 ▸(*a*) (violet triangle label). (*b*), (*c*) and (*d*) are the same multiple lattice image of Fig. 3 ▸(*b*) (cyan triangle label), from which three different lattices were identified. The dashed blue rings correspond to 50, 10 and 7.0 Å in-plane resolution. The magenta cross represents the direct-beam position.

**Figure 5:**
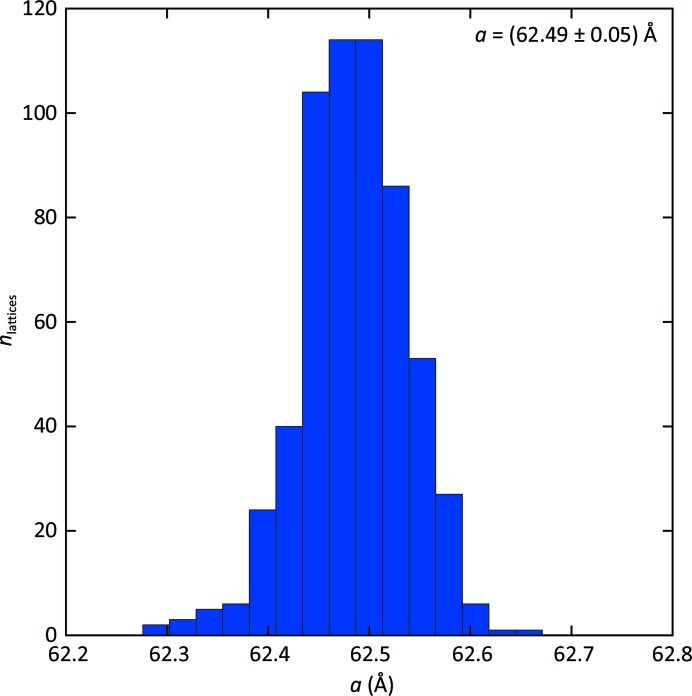
Unit-cell sizes. Histogram of the distribution of the lattice constant *a* refined for 586 lattices.

In the indexable images, 711 lattices were identified and indexed, based on the peak list from *Cheetah* (step 1 in Fig. 2 ▸), and then refined after a search for the most prominent diffraction spots in the diffraction images (step 2 in Fig. 2 ▸), which are marked in red in the example diffraction image of Fig. 4 ▸. This number was reduced to 586 lattices by applying a 28% threshold to the fraction of detected spots in the resolution range down to 4 Å. Fig. 5 ▸ reports the distribution of the refined lattice constant, which shows a very small spread of less than 0.5%.

The distribution of the number of lattices per image at this point of the analysis was as follows: 21.3% of the images delivered a single lattice (see the example in Fig. 4 ▸ *a*), 12.3% delivered two lattices, 4.3% delivered three lattices (see the examples in Figs. 4 ▸ *b*, 4 ▸ *c* and 4 ▸ *d*) and 0.4% delivered four lattices.

**Figure 6:**
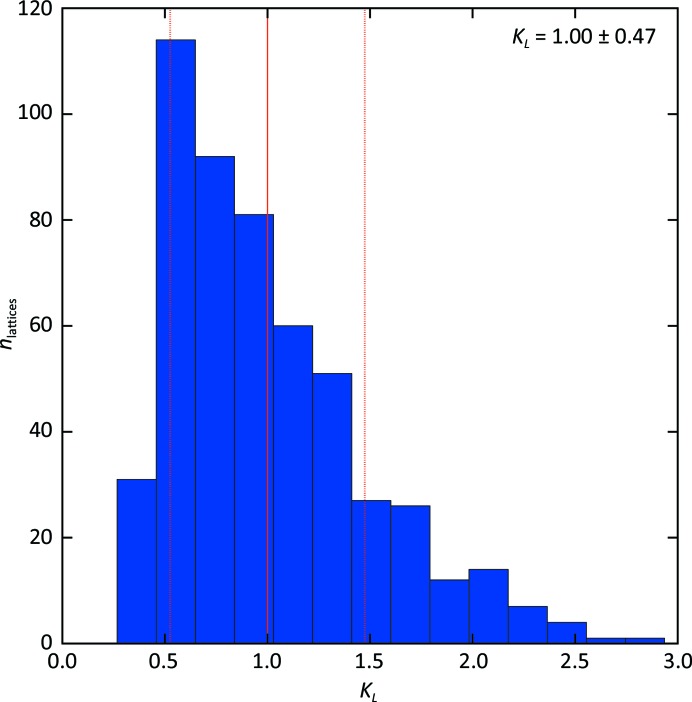
Scale factors. Histogram of the distribution of the lattice-dependent multiplicative scale factors *K_L_* calculated for 521 lattices and imposing an average value of unity.

**Figure 7:**
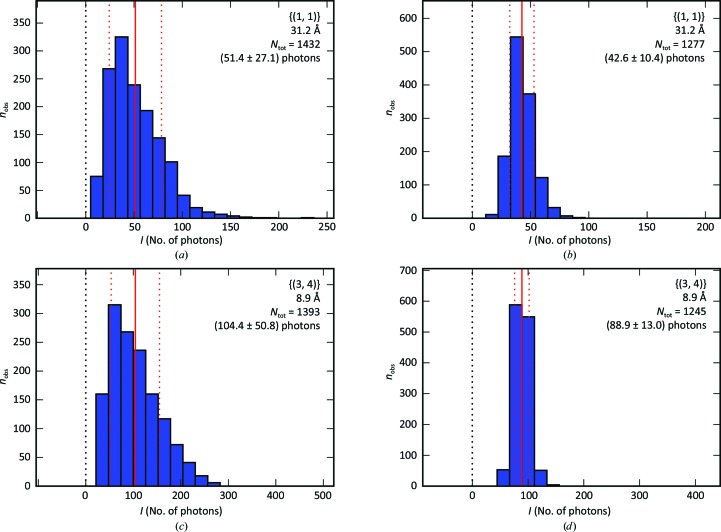
Merged peak intensities. Histograms of the peak intensities of the *p*_3_ reflections {(1, 1)} (*a*, *b*) and {(3, 4)} (*c*, *d*) obtained in step 2 of the data-analysis procedure before rescaling (left column) and after rescaling (right column). The red vertical line represents the average number of photons, and the two dotted red vertical lines delimit the interval within the standard deviation. The in-plane resolution, number of observations, intensity average and intensity standard deviation are reported.

**Figure 8:**
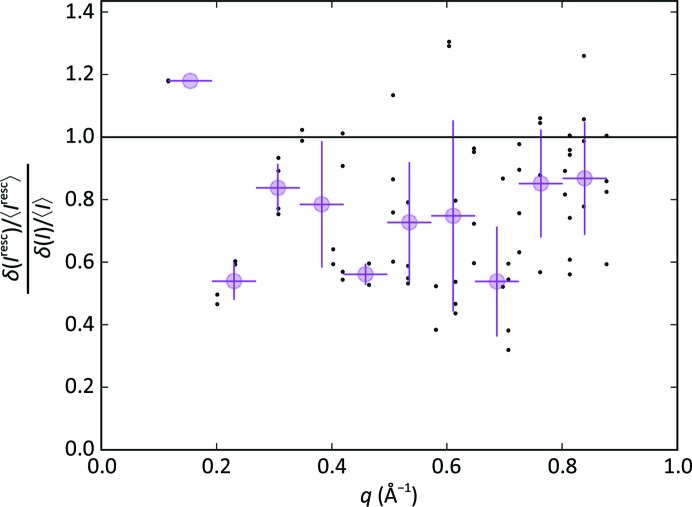
Lattice-scaling effect. Ratio between the peak intensity distribution widths δ(*I*_resc_)/〈*I*_resc_〉 and δ(*I*)/〈*I*〉 after and before scaling, respectively. The ratios are shown as a function of the in-plane momentum transfer of the reflection. Circles in magenta represent the average over resolution bins.

The transformations to solve the indexing ambiguity (step 3 in Fig. 2 ▸) were successfully determined for all lattices except one (585/586). There was full agreement between the outcome of the two methods. 48.4% of the lattices were subjected to the face-reversing transformations *T*_2_ and *T*_3_, which is compatible with the expectation that the two-dimensional crystals are deposited on the support with an equal probability of face orientation. The scaling procedure (step 4 in Fig. 2 ▸) was successful for 521 lattices out of 585 (88.9%). The distribution of the scaling factors is shown in Fig. 6 ▸. Fig. 7 ▸ shows the distribution of the measured peak intensities before and after rescaling for two reflection classes, while Fig. 8 ▸ demonstrates that the width of the distributions is clearly reduced by the rescaling for reflections down to a resolution of 7 Å.

**Figure 9:**
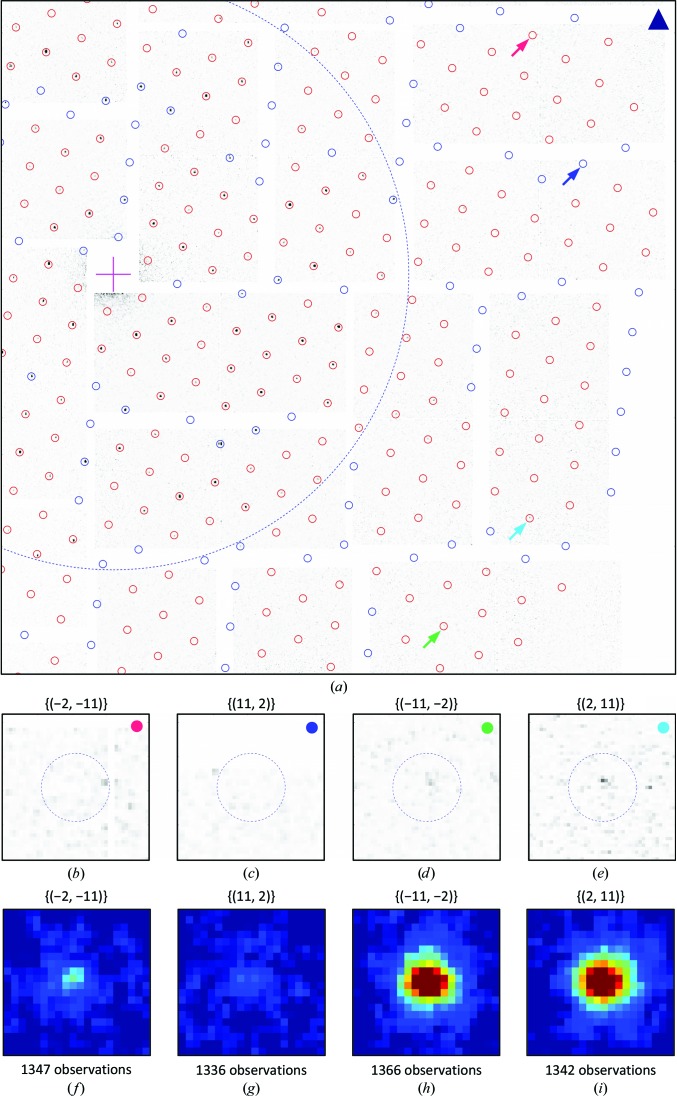
High-resolution data. (*a*) Extension to high resolution of the diffraction image in Figs. 3 ▸(*a*) and 4 ▸(*a*), in which a single lattice was identified. The circles mark the predicted peak positions to 4 Å in-plane resolution. Red circles are valid positions on the detector, while blue circles are invalid positions owing to module gaps or masked pixels. The dashed blue circle corresponds to 7 Å in-plane resolution. The magenta cross represents the direct-beam position. (*b*)–(*e*) Magnifications at the peak positions labelled in (*a*) by an arrow of the corresponding colour. The four reflections are labelled by the indices of the corresponding *p*_3_ reflection {(*h*, *k*)} and have the same in-plane resolution of 4.46 Å. (*f*)–(*i*) Image-sector sums of the four reflections. The number of observations *N* is indicated.

For further evaluation, the 521 lattices and the corresponding images were considered, which correspond to an effective hit rate of 0.54 lattices per image (521/968). Fig. 9 ▸ exemplifies the results of the image-sum procedure (step 5 in Fig. 2 ▸) for the four reflections {(−2, −11)}, {(11, 2)}, {(−11, −2)} and {(2, 11)} at 4.46 Å in-plane resolution. Fig. 9 ▸(*a*) shows the same diffraction image as Figs. 3 ▸(*a*) and 4 ▸(*a*), with the predicted peak positions of the lattice marked down to 4 Å in-plane resolution. Figs. 9 ▸(*b*)–9 ▸(*e*) are magnifications at four predicted peak positions, each belonging to one of the four reflection classes. Figs. 9 ▸(*f*)–9 ▸(*i*) show the corresponding image sums, each resulting from approximately 1300 observations. The enhancement of the signal to noise is nicely visualized, in particular for the reflections {(−2, −11)} and {(11, 2)}, for which the peaks are barely visible in the diffraction images.

**Figure 10:**
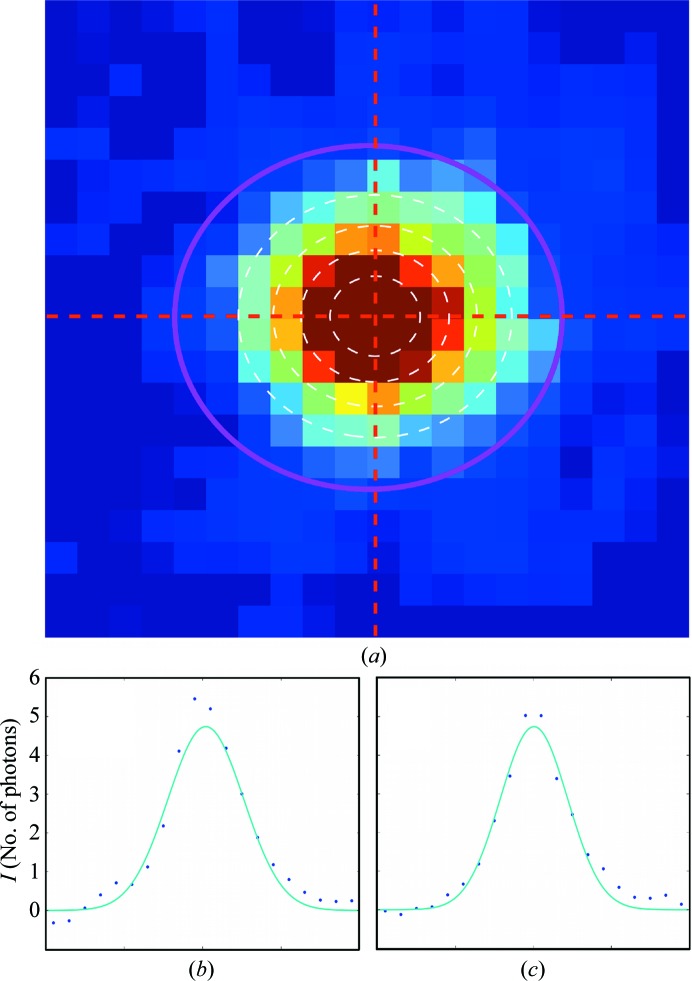
Reflection-peak intensity fit. (*a*) Enlargement of the reflection-image sum {(2, 11)} of Fig. 9 ▸(*i*). The dashed white lines are contour levels of the fitted Gaussian peak function. The magenta ellipse is the integration area, with the semi-axes defined after modelling the width behaviour. (*b*, *c*) Horizontal and vertical sections through the red dotted lines in (*a*). The blue points and cyan lines are the experimental data and the Gaussian fit.

**Figure 11:**
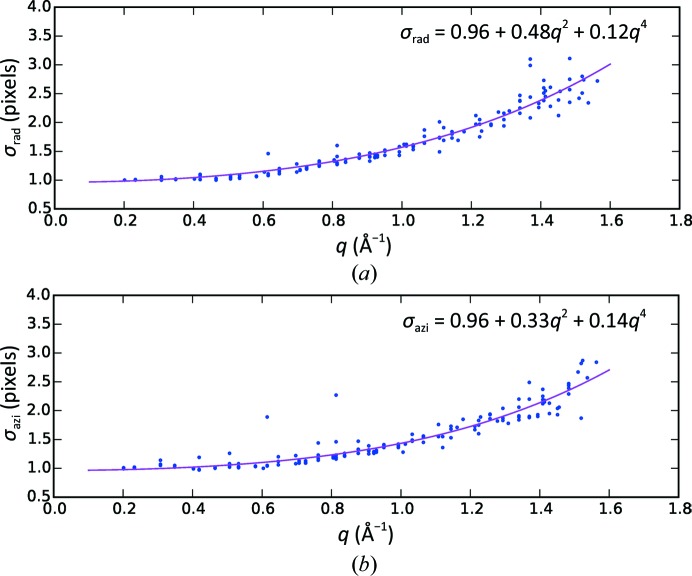
Reflection-peak widths. Widths in (*a*) the radial direction (σ_rad_) and (*b*) the azimuthal direction (σ_azi_) of the reflections, shown as a function of the in-plane momentum transfer *q*. Only the widths of the reflections with an intensity larger than one photon are plotted. The magenta lines represent the biquadratic models indicated in the two panels.

From the per-reflection image sums, we determined the azimuthal and radial widths σ_rad_ and σ_azi_ of the reflections (step 6 in Fig. 2 ▸) by fitting the image sums with a Gaussian peak function (2) (see Fig. 10 ▸). The widths are plotted in Fig. 11 ▸. The step-like artifact at about 7–6 Å in-plane resolution observed after a first iteration (Supplementary Fig. S2) disappeared after correcting the geometry of the outer modules, which allowed the widths to be modelled as a function of the in-plane resolution with the polynomial (3), represented by the magenta curve in the figure.

**Figure 12:**
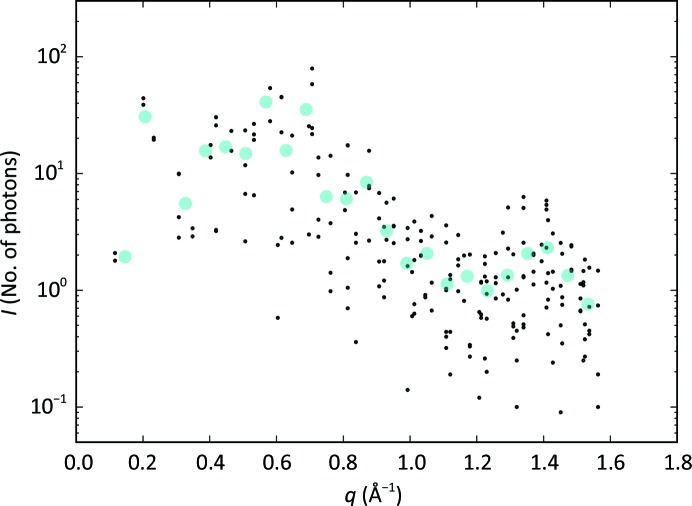
Final reflection intensities. The intensities **I** of the reflections from integration of the image sums, shown as a function of the in-plane momentum transfer *q*. The large cyan circles are resolution-bin averages.

The final reflection intensities were obtained by integration on elliptical areas (step 7 in Fig. 2 ▸), shown by magenta contours in the example in Fig. 10 ▸(*a*). Fig. 12 ▸ presents the intensities of each reflection as a function of the reflection in-plane resolution, as tabulated in Supplementary Table S1. We observe that the typical reflection intensities range from about ten photons per peak at 40 Å resolution to one photon per peak at 4 Å resolution.

**Figure 13:**
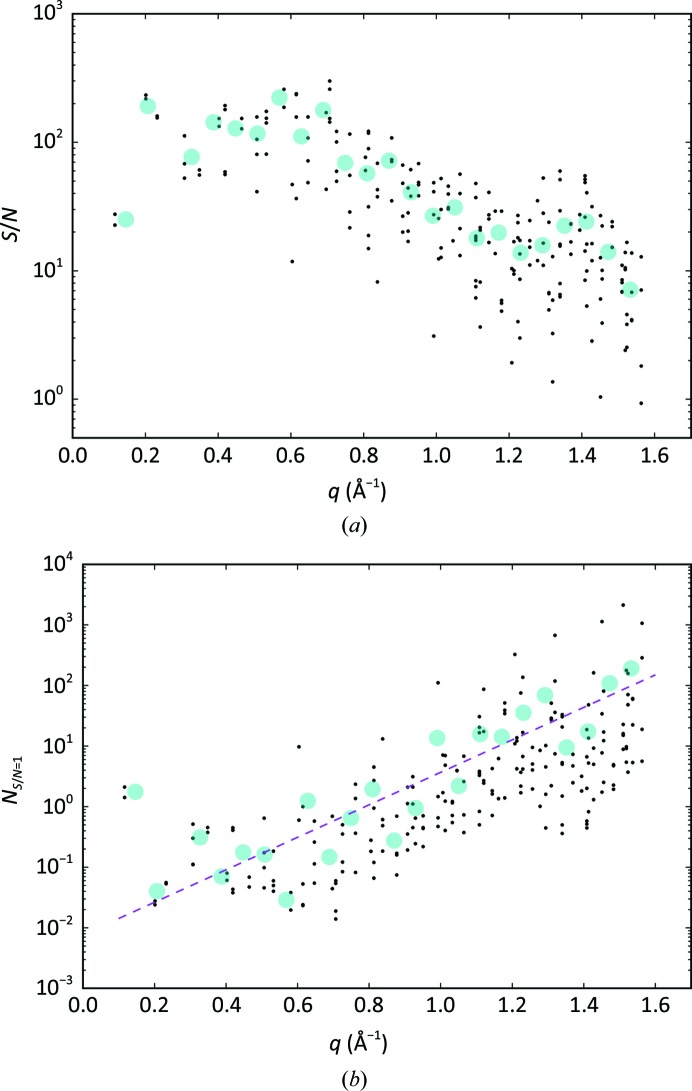
Signal to noise. (*a*) Signal-to-noise ratio S/N of the reflection intensity. (*b*) Estimated number of observations of each reflection to achieve a signal-to-noise value of unity (*N*_S/N = 1_), with the dashed magenta line representing the best exponential fit. In both panels the values are shown as a function of the in-plane momentum transfer of the reflection. The large cyan dots are resolution-bin averages.

The per-reflection S/N values were calculated following (6) and are shown in Fig. 13 ▸(*a*). The S/N decreases from 100 to the order of 10 from low to 4 Å in-plane resolution. The factor of ten decrease, which is larger than the expected 10^1/2^ from Poisson noise, reflects the contributions from the image-sum background that become more relevant at higher resolution. We checked that the S/N scales as expected as 1/*N* ^1/2^ by evaluating reduced data sets with ten and 100 lattices (Supplementary Fig. S3). Fig. 13 ▸(*b*) reports the number of observations *N* that are necessary to achieve an S/N equal to unity at a given resolution, calculated for each reflection according to (7). The magenta dashed line in the figure represents the overall trend modelled as an exponential. At 4 Å resolution the required number of observation is close to 200.

**Figure 14:**
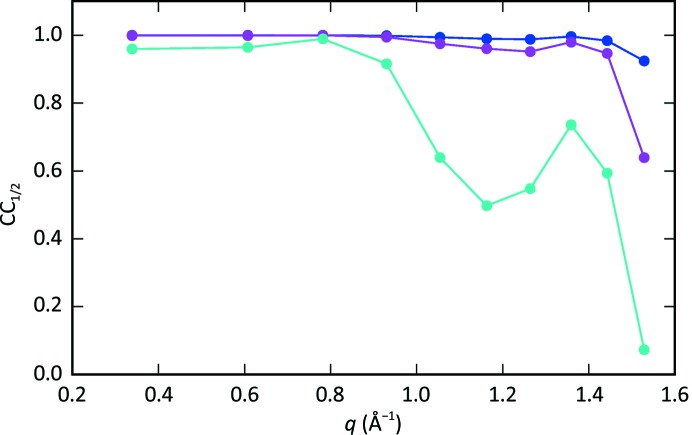
Split correlation coefficient CC_1/2_ obtained using the full data set of 586 lattices (blue), 100 lattices (magenta) and ten lattices (cyan). The values are shown as a function of the in-plane momentum transfer *q* in the centre of the resolution bin. Each resolution bin includes 22 reflections and each point is the average over ten different random splittings of the data set.

Fig. 14 ▸ shows the split correlation coefficient CC_1/2_ in resolution bins as a function of resolution, calculated for the full data set of 521 lattices, as well as for reduced data sets consisting of 100 and ten randomly chosen lattices. With increasing number of lattices, the correlation coefficient approaches 1 in all resolution bins.

## 4. Discussion   

To analyze the two-dimensional crystal diffraction patterns, we mostly implemented concepts from serial single-shot three-dimensional protein crystallo­graphy, such as lattice identification, lattice-parameter refinement and indexing-ambiguity solution. We relied on previous knowledge of the two-dimensional space group; however, the protocol can easily be extended by establishing the crystal symmetry using low-resolution spots.

To fully exploit the available data to the highest possible resolution, we implemented the non-conventional image-sum method that enhances the signal-to-noise ratio of the measured reflection intensities. This approach was crucial, in particular for refining the detector geometry *a posteriori*. Our quality indicators show that with the full data set the resolution corresponding to the 4 Å limit given by the detector area could be achieved. Similarly, our evaluations provide a method to predict the number of reflection observations necessary to achieve a certain resolution. For example, for 4 Å in-plane resolution (3.95 Å three-dimensional resolution) the requirement is for about 200 observations. This number increases to about 500 at 3.5 Å in-plane resolution, as obtained by extrapolating from the experimental data.

The data discussed in this article were recorded at zero tilt angle. In this configuration, for each Bragg rod (*h*, *k*) the value of the diffraction intensity can only be measured at two opposite reciprocal-space coordinates ±*q_z_*(*h*, *k*) along the rod, whereby the point with negative *q_z_* value is the Friedel mate of a point of another rod with positive *q_z_* value. Because of the curvature of the Ewald sphere, *q_z_*(*h*, *k*) is not vanishing, therefore not even the reconstruction of a two-dimensional density projection is possible. If the orientation of the two-dimensional crystals on the sample support is random, then recording data with a tilted sample chip allows the continuous sampling of *q_z_* in a range along each rod, yielding a genuine three-dimensional data set, which is however affected by a missing data wedge, as in electron-microscopy and diffraction approaches (Unwin & Henderson, 1975*b* ▸). Most of the key algorithms developed for the present analysis, such as lattice identification, peak search, lattice-parameter refinement, indexing-ambiguity solution and lattice scaling, are implemented to treat tilted data. The procedure to reconstruct intensities along Bragg lines in reciprocal space will be detailed in a separate article, in which we analyze and merge a few data sets collected at various tilt angles but with lower redundancy and lower detector-edge resolution than the data set in the present paper, and we address the key point of showing that the three-dimensional intensity data set is meaningful.

The single-layer assembly implies less steric hindrance in general compared with three-dimensional crystals and opens up the possibility of observing large-scale dynamics in a pump–probe experiment, in which an optical pump triggers a structural change that is probed, after a suitable time delay, by a femtosecond X-ray pulse. In this respect, it becomes essential to optimize the methods for sample preservation in the vacuum of the experimental chamber. A viable alternative to sugar-embedding may be to ‘sandwich’ the sample within two thin membranes, for example of silicon nitride, as we tested preliminarily during the November 2013 beamtime, or of graphene, as originally developed for use in electron diffraction (Gyobu *et al.*, 2004 ▸) and then modified for two-dimensional SFX (Frank *et al.*, 2014 ▸). Owing to loose crystal packing and femtosecond time resolution, two-dimensional SFX is complementary to three-dimensional SFX and to electron-based methods, respectively, and has the potential to provide information on dynamics in systems that allow crystallization in two dimensions.

## 5. Conclusions   

The results of the experiments demonstrate that the measured two-dimensional bacteriorhodopsin crystals diffract to at least 4 Å resolution and that the diffraction signal can be reliably measured at this resolution from less than 100 images obtained using the setup at the CXI beamline of the LCLS X-ray free-electron laser. The very low signal intensities required the implementation of analysis methods relying on image sums. The resolution limit comes only from the experiment geometry.

Ultimately, the overall outcome brings us towards near-atomic resolution two-dimensional crystallography and to pump–probe studies of the structural dynamics of membrane proteins in a loose-packing environment, where large-scale movements are allowed.

## Supplementary Material

Pseudo-codes, Supplementary Figs. S1, S2 and S3 and Supplementary Table S1.. DOI: 10.1107/S2052252517017043/ec5006sup1.pdf

## Appendix A Indexing-ambiguity solution by linear correlation

We formulated the linear correlation method for solving the indexing ambiguity in general terms in view of also processing data with nonzero tilt angle. Linear correlations have already been used in the three-dimensional SFX detwinning approach to cluster patterns with the same indexing mode (Brehm & Diederichs, 2014 ▸), as implemented in *CrystFEL* (White *et al.*, 2016 ▸). The method implemented here does not involve pattern clustering, but rather a direct comparison of patterns in the different indexing scenarios. The details are given below.

### A1. Determination of the indices transformation

The transformation of indices *T*_*S*−*L*_ that is required to make a lattice *L* in the data set compatible in indexing with a randomly extracted reference lattice or ‘seed’ *S* was determined as follows. A linear correlation coefficient between intensities from the seed and those from lattice *L* was calculated as described in §[Sec seca2]A2 in the assumption of each of the possible transformations *T_i_*:

(i) *T*_0_(*h*, *k*, *q_z_*) = (*h*, *k*, *q_z_*),
(ii) *T*_1_(*h*, *k*, *q_z_*)) = (−*h*, −*k*, *q_z_*),
(iii) *T*_2_(*h*, *k*, *q_z_*) = (*k*, *h*, −*q_z_*) ≡ (−*k*, −*h*, *q_z_*),
(iv) *T*_3_(*h*, *k*, *q_z_*) = (−*k*, −*h*, -*q_z_*) ≡ (*k*, *h*, *q_z_*).

The largest of these coefficients is that related to the most likely transformation. The reliability of the determination of *T*_*S*−*L*_ was assessed by randomly extracting a large number (*e.g.* 100) of lattices *L*′, for each of which the expression

was evaluated. When the three transformations involved are correctly determined, this expression equals identity. We considered the identification of *T*_*S*–*L*_ to be reliable if the expression above was equal to identity for at least 70% of the lattices *L*′.

The procedure was repeated extracting different seeds *S_j_* and the consistency of the results was checked. The transformation  required to make the indexing of different seeds compatible was determined and the transformation of all lattices (for which a value of  had been reliably determined) with respect to the same seed was calculated:  = .

### A2. Calculation of the linear correlation coefficients

The linear correlation coefficient relating the intensities from two lattices *L*_1_ and *L*_2_ was calculated as follows, with the assumption of each of the transformations *T_i_* defined in §A1. For each indexed spot (*h*, *k*, *q_z_*) in *L*_1_, a search was carried out through *L*_2_ looking for spots with indices *T_i_*(*h*, *k*, *q_z_*). While the four transformations formulated above leave the value of *q_z_* unaltered, it was necessary to relax the condition *q_z_*(*L*_1_) = *q_z_*(*L*_2_) in the search across *L*_2_, setting a nonzero upper limit for the tolerated difference, since the method was written in a general way to also treat data from tilted-stage measurements.

Both *p*3 lattice symmetry and Friedel symmetry were considered in the search described above. This is important in the case of nonzero tilt, where the number of pairs entering the correlation calculation is relatively small. In practice, for each transformation *T_i_*, (*h*, *k*, *q_z_*) in *L*_1_ was matched to six equivalent spots in *L*_2_ (when measured),

and their Friedel mates. Here  indicates the transformed indices  and  ≃ *q_z_*.

## Appendix B Indexing-ambiguity solution by the expansion–maximization–compression algorithm

An alternative method for resolving the indexing ambiguity is the use of the expansion–maximization–compression (EMC) algorithm (Loh & Elser, 2009 ▸), which was originally devised for determining the orientation of single-particle diffraction patterns. Less well known is that the same technique can be modified and adapted for solving the ambiguity in the indexing of serial crystallography (SX) data. The sparse images are replaced with reflection intensities, the search space of angles become the set of possible reindexing operators (defined in §[Sec sec2.3.2]2.3.2 in the case of plane group *p*3) and the three-dimensional compressed model becomes the final set of merged intensities. The Poisson probability operator can be retained for use with very weak patterns consisting of single photon counts (Philipp *et al.*, 2012 ▸; Ayyer *et al.*, 2015 ▸), while for stronger patterns of varying intensity this can be replaced with a cross-correlation metric, thereby obviating the need to determine the relative scaling of each pattern. The modified EMC algorithm becomes the following.

(i) Create an initial model for reflection intensities based on either random start or on the average value of observations merged according to the original (ambiguous) indexing.
(ii) Compute the cross-correlation of each individual pattern with the model for each operation that leaves diffraction positions unaltered (reindexing operation). This includes the point-group symmetry of the lattice and, for two-dimensional crystals in *p*3, the possibility of an in-plane π rotation, the face reversal of the crystal and their combination.
(iii) Assign the pattern-reindexing transformation to that with the highest cross-correlation with the current model.
(iv) Merge all patterns in their newly assigned indexing, update the current model and repeat from step (ii).

The known point-group symmetry of the crystal can be applied at this stage, if it is known, to merge symmetry-equivalent reflections. In practice, we found that assignments were stable and nonchanging after less than ten iterations of the loop. Observations are merged based on the final re­indexing assignment obtaining the set of reflection intensities used for structure determination. The same procedure works very well for solving the indexing ambiguity in three-dimensional SX data in addition to the two-dimensional SFX case studied here.

## Appendix C Lattice-intensity rescaling

The multiplicative factor *K*_*L*−*S*_ required to scale the intensities from a lattice *L* to those from a randomly extracted seed *S* was calculated by linear least-squares fitting,

where  ≃ *q_z_* and  are the *p*3-transformed  and their Friedel mates.

To verify the reliability of this estimate of *K*_*L*−*S*_ we adopted a method analogous to that described in §[Sec seca1]A1. A large number (*e.g.* 100) of lattices *L*′ was extracted, for each of which the expression

was evaluated. We considered the value of *K*_*L*−*S*_ to be acceptable if a value close to 1 (between 0.75 and 1.25) in the expression above was obtained with at least 70% of the lattices *L*′. The procedure was repeated extracting different seeds and the consistency of the results was checked. The average value of scaled, equivalent reflections was used as a preliminary indication of intensity.

## Appendix D Determination of translational corrections to detector-module positions

Translational corrections *t^m^_x_* and *t^m^_y_* to the position of each detector module *m* were determined as follows. For each reflection class {(*h*, *k*)} with sufficient intensity, a partial sum was calculated by summing image sectors belonging to module *m*. The partial sum was fitted using a two-dimensional Gaussian function and the distance between the refined centre coordinates of the fitting function and the predicted spot position was considered to be an estimate *t^m^*_*x*,{(*h*,*k*)}_ and *t^m^*_*y*,{(*h*,*k*)}_ of the translational error. Consistent results were found repeating the procedure for all orbits {(*h*, *k*)} with spots on a given module, and sufficient intensity to obtain a meaningful fit. The translations for a module *m* were calculated as the average on all reflection classes {(*h*, *k*)} of *t^m^*_*x*,{(*h*,*k*)}_ and *t^m^*_*y*,{(*h*,*k*)}_. The pseudo-code describing this procedure is reported in the Supporting Information.
