# Supplementary material for: Resolution extension by image summing in serial femtosecond crystallography of two-dimensional membrane-protein crystals
Source: IUCrJ. 2018 Jan 1;5(Pt 1):103–17. doi: 10.1107/S2052252517017043 (PMC5755582; doi:10.1107/S2052252517017043)
Supplement: Supplementary file 1 [file m-05-00103-sup1.pdf]

IUCrJ

Volume 5 (2018)

Supporting information for article:

Resolution extension by image summing in serial femtosecond crystallography of two-dimensional membrane-protein crystals

Cecilia M. Casadei, Ching-Ju Tsai, Anton Barty, Mark S. Hunter, Nadia A. Zatsepin, Celestino Padeste, Guido Capitani, W. Henry Benner, Sébastien Boutet, Stefan P. Hau-Riege, Christopher Kupitz, Marc Messerschmidt, John I. Ogren, Tom Pardini, Kenneth J. Rothschild, Leonardo Sala, Brent Segelke, Garth J. Williams, James E. Evans, Xiao-Dan Li, Matthew Coleman, Bill Pedrini and Matthias Frank

Pseudo-code describing the determination of detector module translations.

for *module in modules* **do**
    for *orbit in orbits* **do**
        if $I_{orbit} > threshold$ **then**
            $sum_{orbit,module} \leftarrow 0$
            for *lattice in lattices* **do**
                for *spot in spots* **do**
                    if $spot \in orbit$ **then**
                        if $spot \in module$ **then**
                            $sector \leftarrow \text{extractSector}(spot,\ lattice)$
                            $sector \leftarrow \text{rotateSector}(sector)$
                            $sum_{orbit,module} \leftarrow sum_{orbit,module} + sector$
                        end
                    end
                end
            end
            $sum_{orbit,module} \leftarrow \text{backgroundSubtraction}(sum_{orbit,module})$
            $t_x^{orbit,module}, t_y^{orbit,module} \leftarrow \text{gaussFit}(sum_{orbit,module})$
        end
    end
    $t_x^{module} \leftarrow \left\langle t_x^{orbit,\, module} \right\rangle$
    $t_y^{module} \leftarrow \left\langle t_y^{orbit,\, module} \right\rangle$
end

Pseudo-code describing the procedure for image averaging.

for *orbit in orbits* **do**
    $sum_{orbit} \leftarrow 0$
    for *module in modules* **do**
        $sum_{orbit,module} \leftarrow 0$
        for *lattice in lattices* **do**
            for *spot in spots* **do**
                if $spot \in orbit$ **then**
                    if $spot \in module$ **then**
                        $sector \leftarrow \text{extractSector}(spot,\ lattice)$
                        $sector \leftarrow \text{scaleSector}(sector)$
                        $sector \leftarrow \text{rotateSector}(sector)$
                        $sum_{orbit,module} \leftarrow sum_{orbit,module} + sector$
                    end
                end
            end
        end
        $sum_{orbit,module} \leftarrow \text{recenter}(sum_{orbit,module}, t_x^{module}, t_y^{module})$
        $sum_{orbit} \leftarrow sum_{orbit} + sum_{orbit,module}$
    end
    $sum_{orbit} \leftarrow \text{backgroundSubtraction}(sum_{orbit})$
    $sum_{orbit} \leftarrow sum_{orbit}/N_{terms}$
    $\sigma_x^{orbit}, \sigma_y^{orbit} \leftarrow \text{gaussFit}(sum_{orbit})$
end

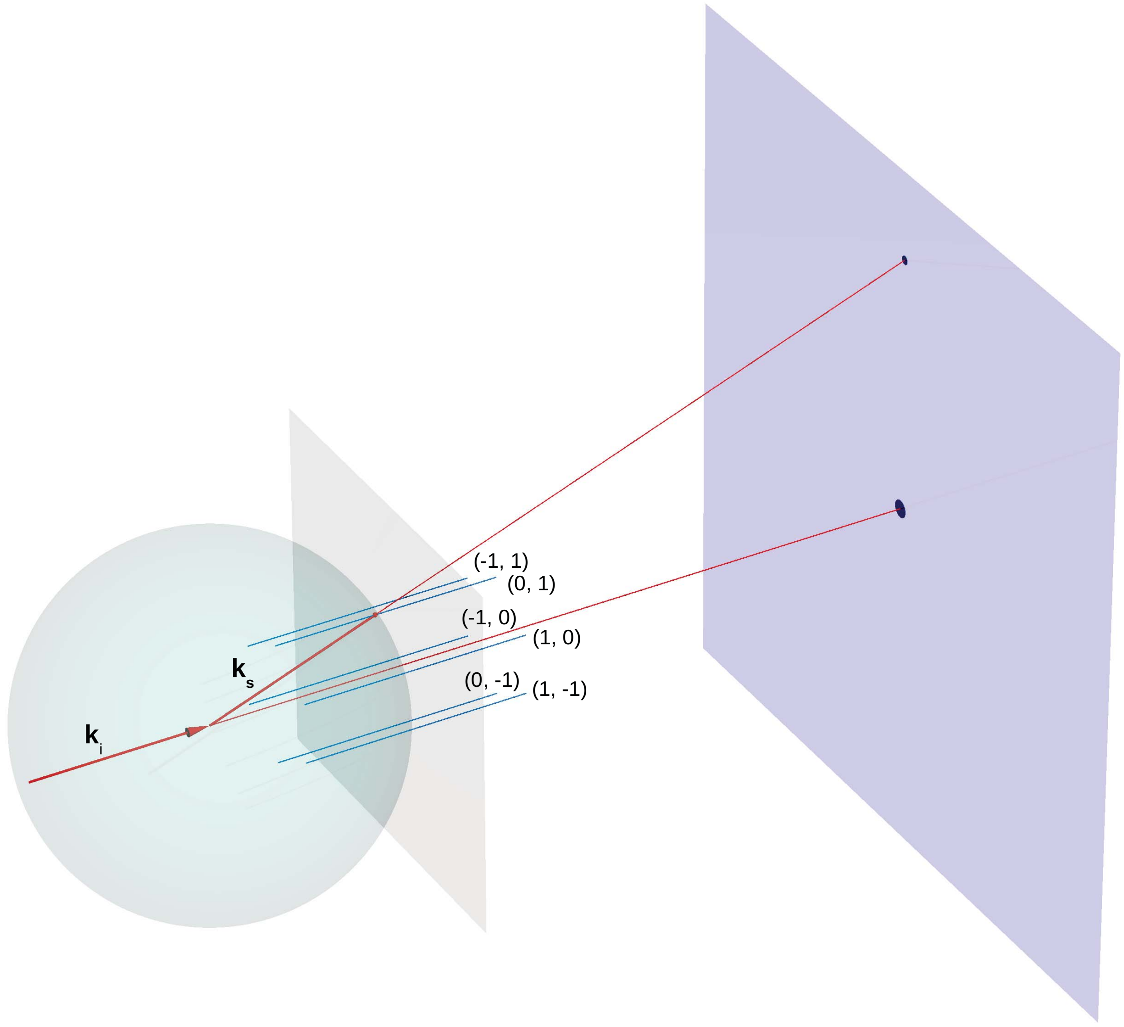

Fig. S1. *Generation of diffraction spots from two-dimensional crystals.* In a system with periodicity in two dimensions the diffraction condition is fulfilled at the intersection between the Ewald sphere and Bragg rods (h, k).

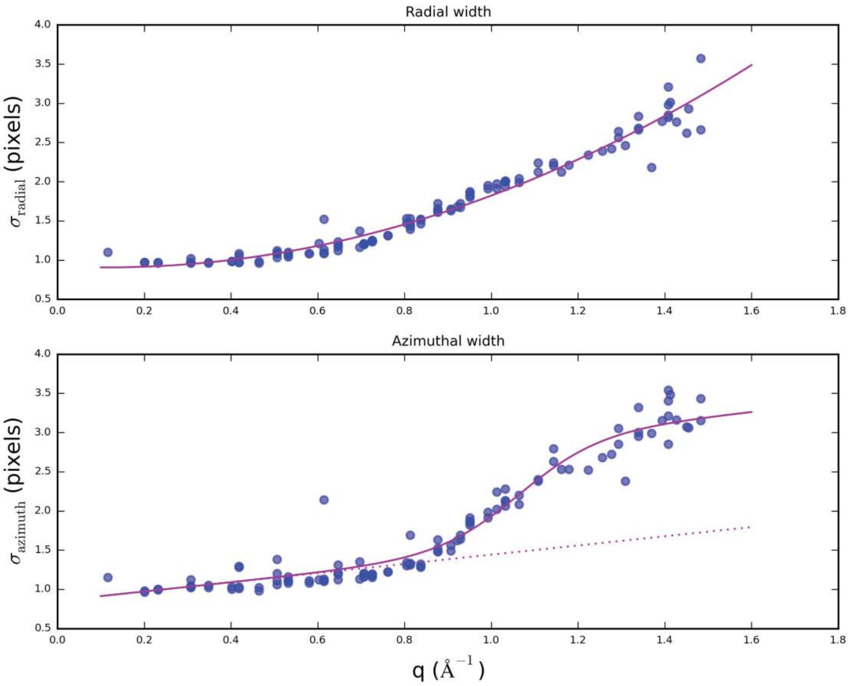

Fig. S2. *Diffraction spot shape* Radial and azimuthal widths as a function of 2D-resolution obtained by gaussian fitting of image sums. No translational correction to detector module positions was applied. Unique reflections with intensity above 2 photons were considered. The radial width shows a quadratic behaviour while the azimuthal width is linear at low resolution and deviates with a sigmoid shape at high q-values.

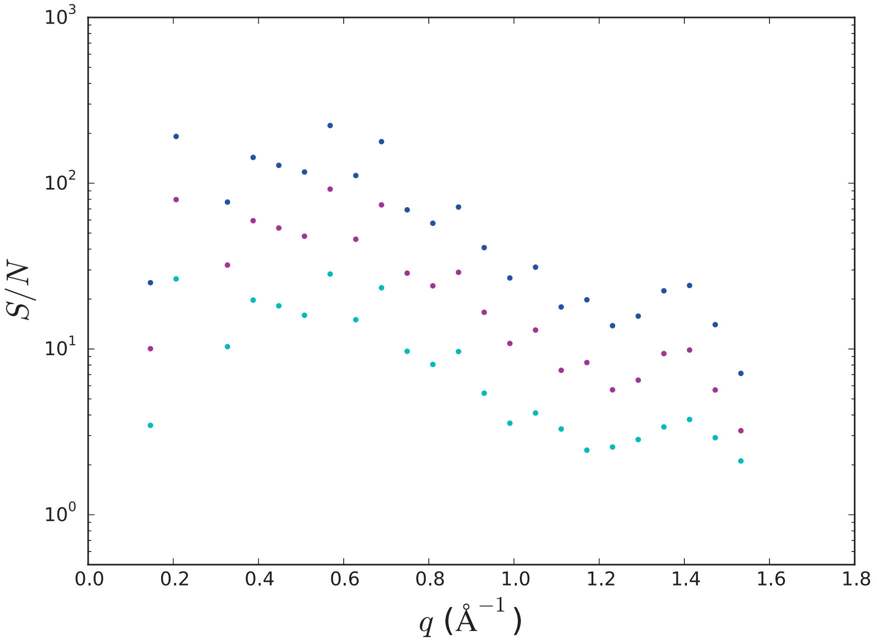

Fig. S3. *Signal to noise ratio calculated using portions of the dataset.* Signal to noise ratio in image sums as a function of 2D-resolution, using the full dataset of 586 lattices (blue), 100 lattices (magenta) and 10 lattices (cyan). Resolution bins have the same width in q.

Table S1. Reflection intensities obtained by integration of the sector sums over elliptical areas.

h	k	I (photons)
-13	0	0.67
-12	-2	0.38
-12	-1	2.05
-12	0	1.77
-11	-4	0.74
-11	-3	2.43
-11	-2	5.38
-11	-1	0.50
-11	0	0.91
-10	-5	0.72
-10	-4	1.09
-10	-3	1.43
-10	-2	0.82
-10	-1	1.69
-10	0	0.81
-9	-6	0.25
-9	-5	1.04
-9	-4	2.54
-9	-3	0.85
-9	-2	0.34
-9	-1	1.03
-9	0	0.87
-8	-7	1.14
-8	-6	3.99
-8	-5	0.10
-8	-4	0.20
-8	-3	1.63
-8	-2	1.16
-8	-1	3.41
-8	0	5.62
-7	-8	0.85
-7	-7	4.91
-7	-6	2.03
-7	-5	0.58
-7	-4	0.43
-7	-3	1.98
-7	-2	3.52
-7	-1	7.83
-7	0	0.70
-6	-9	1.44

Continuation of Table S1		
h	k	I (photons)
-6	-8	1.40
-6	-7	0.39
-6	-6	0.65
-6	-5	0.44
-6	-4	1.81
-6	-3	1.21
-6	-2	6.87
-6	-1	1.41
-6	0	3.00
-5	-10	0.45
-5	-9	1.45
-5	-8	1.01
-5	-7	1.16
-5	-6	0.32
-5	-5	0.61
-5	-4	4.12
-5	-3	17.45
-5	-2	4.02
-5	-1	21.08
-5	0	28.01
-4	-11	0.20
-4	-10	0.10
-4	-9	6.29
-4	-8	1.19
-4	-7	0.19
-4	-6	0.63
-4	-5	1.08
-4	-4	4.85
-4	-3	24.47
-4	-2	44.87
-4	-1	26.55
-4	0	23.10
-3	-11	1.46
-3	-10	2.06
-3	-9	1.15
-3	-8	1.84
-3	-7	2.00
-3	-6	3.50
-3	-5	1.05
-3	-4	58.05
-3	-3	2.44
-3	-2	2.62

Continuation of Table S1		
h	k	I (photons)
-3	-1	3.21
-3	0	2.89
-2	-12	0.26
-2	-11	1.16
-2	-10	5.11
-2	-9	0.32
-2	-8	0.67
-2	-7	3.56
-2	-6	3.04
-2	-5	9.69
-2	-4	2.81
-2	-3	6.68
-2	-2	17.51
-2	-1	9.96
-2	0	20.22
-1	-12	0.87
-1	-11	5.05
-1	-10	1.94
-1	-9	3.59
-1	-8	1.62
-1	-7	7.47
-1	-6	3.75
-1	-5	2.55
-1	-4	19.36
-1	-3	30.24
-1	-2	2.82
-1	-1	44.02
-1	0	1.79
1	0	2.08
1	1	38.69
1	2	4.22
1	3	25.79
1	4	21.56
1	5	4.91
1	6	14.16
1	7	2.66
1	8	0.14
1	9	2.56
1	10	1.30
1	11	0.48
1	12	0.35
2	0	19.42

Continuation of Table S1		
h	k	I (photons)
2	1	9.86
2	2	13.71
2	3	23.37
2	4	22.49
2	5	13.69
2	6	0.36
2	7	6.09
2	8	2.89
2	9	0.27
2	10	1.29
2	11	5.86
2	12	0.51
3	0	3.39
3	1	3.28
3	2	11.75
3	3	0.57
3	4	79.11
3	5	1.88
3	6	0.87
3	7	3.22
3	8	0.98
3	9	2.08
3	10	1.12
3	11	2.37
4	0	15.62
4	1	6.50
4	2	45.23
4	3	21.71
4	4	6.85
4	5	6.80
4	6	0.76
4	7	1.24
4	8	0.93
4	9	1.31
4	10	2.53
4	11	0.12
5	0	53.76
5	1	10.19
5	2	2.87
5	3	9.72
5	4	1.76
5	5	1.43

Continuation of Table S1		
h	k	I (photons)
5	6	1.00
5	7	1.18
5	8	0.25
5	9	3.05
5	10	1.55
6	0	25.36
6	1	0.98
6	2	2.55
6	3	1.77
6	4	3.87
6	5	0.40
6	6	0.12
6	7	0.51
6	8	0.42
6	9	1.10
7	0	17.36
7	1	15.63
7	2	2.53
7	3	2.65
7	4	1.35
7	5	0.62
7	6	0.49
7	7	2.31
7	8	0.66
8	0	2.70
8	1	2.73
8	2	4.32
8	3	2.96
8	4	0.58
8	5	0.45
8	6	0.83
8	7	0.86
9	0	0.91
9	1	1.01
9	2	2.03
9	3	1.29
9	4	1.30
9	5	0.23
9	6	1.17
10	0	1.98
10	1	0.26
10	2	2.27

Continuation of Table S1		
h	k	I (photons)
10	3	2.00
10	4	0.50
10	5	0.42
11	0	3.11
11	1	0.61
11	2	0.70
11	3	1.52
11	4	1.48
12	0	2.46
12	1	0.75
12	2	1.84
13	0	-0.06
